# Residents in difficulty: a mixed methods study on the prevalence, characteristics, and sociocultural challenges from the perspective of residency program directors

**DOI:** 10.1186/s12909-016-0596-2

**Published:** 2016-02-22

**Authors:** Mette K. Christensen, Lotte O’Neill, Dorthe H. Hansen, Karen Norberg, Lene S. Mortensen, Peder Charles

**Affiliations:** Centre for Health Sciences Education, Aarhus University, Palle Juul-Jensens Boulevard 82, 8200 Aarhus N, Denmark; Northern Postgraduate Medical Training Region Secretariat, Skottenborg 26, 8800 Viborg, Denmark; Region Hospital Randers, Skovlyvej 1, 8930 Randers Ø, Denmark

**Keywords:** Postgraduate training, Problem residents, Residents in difficulty, Mixed methods study, Residency program directors, Pierre Bourdieu, Workplace culture

## Abstract

**Background:**

The majority of studies on prevalence and characteristics of residents in difficulty have been conducted in English-speaking countries and the existing literature may not reflect the prevalence and characteristics of residents in difficulty in other parts of the world such as the Scandinavian countries, where healthcare systems are slightly different. The aim of this study was to examine prevalence and characteristics of residents in difficulty in one out of three postgraduate medical training regions in Denmark, and to produce both a quantifiable overview and in-depth understanding of the topic.

**Methods:**

We performed a mixed methods study. All regional residency program directors (*N* = 157) were invited to participate in an e-survey about residents in difficulty. Survey data were combined with database data on demographical characteristics of the background population (*N* = 2399) of residents, and analyzed statistically (Chi-squared test (*Χ*^2^) or Fisher’s exact test). Secondly, we performed a qualitative interview study involving three focus group interviews with residency program directors. The analysis of the interview data employed qualitative content analysis.

**Results:**

73.2 % of the residency program directors completed the e-survey and 22 participated in the focus group interviews. The prevalence of residents in difficulty was 6.8 %. We found no statistically significant differences in the prevalence of residents in difficulty by gender and type of specialty. The results also showed two important themes related to the workplace culture of the resident in difficulty: 1) belated and inconsistent feedback on the resident’s inadequate performance, and 2) the perceived culturally rooted priority of efficient patient care before education in the workplace. These two themes were emphasized by the program directors as the primary underlying causes of the residents’ difficulty.

**Conclusions:**

More work is needed in order to clarify the link between, on the one hand, observable markers of residents in difficulty and, on the other hand, immanent processes and logics of practice in a healthcare system. From our perspective, further sociological and pedagogical investigations in educational cultures across settings and specialties could inform our understanding of and knowledge about pitfalls in residents’ and doctors’ socialization into the healthcare system.

## Background

International literature reports that 3–10 % of doctors in postgraduate medical education struggle to comply with educational requirements and occupational adaptation [1–4]. These residents in difficulty^1^ often require further support or an extended employment period to achieve the required competencies in their specialist training [5]. In addition, residents in difficulty risk posing a liability to patients’ healthcare [6, 7], and, for this reason, it is important that the healthcare system considers, recognizes and eventually takes precautionary measures against the causes of the difficulties [8, 9]. In order to ensure successful residency training, it is important to identify and define residents in difficulty [7]. Helping residents in difficulty is a complicated endeavor for medical teachers, clinical supervisors, and residency program directors [10], because residents in difficulty constitute a multifarious group [5]. Some residents in difficulty fail to demonstrate the expected medical competences at the right time and in the right situations; some have an illness or suffer from psychological distress; others struggle to fit in with the culture in a specific ward and perhaps even experience bullying at work [12]. Whilst several studies have contributed to our knowledge about residents in difficulty in North America [1, 4, 6, 11] and the United Kingdom [2, 13, 14], it could be argued that these studies may not reflect the prevalence and characteristics of residents in difficulty in other parts of the world such as the Scandinavian countries where healthcare systems are slightly different [15]. To the best of our knowledge, no empirical studies exist on residents in difficulty in Denmark, Norway, or Sweden, and we lack sufficient evidence of the prevalence and characteristics of residents in difficulty in these countries. Furthermore, the main part of studies on residents in difficulty seems to be domain-specific investigations of one specialty, for example, internal medicine [16] and surgery [12, 17]. Only very few studies are cross-specialty studies [5]. This may call for more studies across specialties in the hospitals in order to investigate possible similarities or differences. Lastly, this field of research seem to be a mix of practical experiences [13, 18–21], literature reviews [22], quantitative retrospective surveys [1, 6, 12, 23, 24], and qualitative studies [25–29], which indicates that it is still burgeoning and searching to find its key concepts and preferred research methods.

In this paper, we will examine the prevalence and characteristics of residents in difficulty in Denmark across hospital specialties. The main research questions addressed are:

- What are the demographic characteristics of the residents in difficulty?
- What are the reported behavioral problems and the presumed contributory causes of the residents in difficulty as they are perceived by the residency program directors?

We will begin the paper with a description of the methods and the context of the study. Then we will present the results of the data collection, and finally we will discuss the results in relation to two particular issues: 1) the personal characteristics of residents in difficulty; and 2) malfunctioning of the healthcare system as an educational arena. The paper is based on the results from a mixed methods study across all specialties in hospitals in the region sampled. Although Denmark is a small country, the results from this study may shed light on the apparent discrepancy between two components of residency training in hospitals: the strong emphasis on individual attributes as explanations for the resident’s difficulty and the culturally rooted anticipated social relationship between the resident and the workplace environment.

## Methods

The study was designed as a mixed methods study. The study comprised of an e- survey, a database study, and a qualitative interview study. A mixed methods strategy is appropriate when a researcher intends to approach a research question or a topic from different angles [30]. In this study, we intended to examine both the prevalence and the characteristics of residents in difficulty in one out of three postgraduate medical training regions in Denmark, and we intended to produce both a quantifiable overview and in-depth understanding of the topic.

### Context

Postgraduate medical education in Denmark is governed by the Danish National Board of Health and amounts to 8–9 years of enrolment in a residency program. The program includes two parts: 1) 1 year in basic programs in which the resident is employed in different clinical departments for a period of 6 months in each department; and 2) 5–6 years in a specialist program in which the resident is employed in alternating clinical departments for a period of 6–36 months in each. Most residents start at a regional hospital and then after 12–24 months they shift to a university hospital department. A typical residency program introduces the resident to a variety of clinical specialties and after 2 years it centres around one specialty. The completion of the specialist program, including approved competencies and mandatory courses, authorizes the resident to practice as a specialist doctor. The Danish healthcare system is dependent on most residents in Denmark eventually becoming specialist doctors and, accordingly, most residents anticipate reaching a specialist doctor position after the completion of the residency program. Because the residency program takes place in clinical departments, each participating department is obliged to nominate one of the clinical consultants in the department to direct and manage the residency program in the department [31]. These residency program directors are responsible for: 1) providing educational programs for a highly diverse group of residents (basic, introductory, or specialist programs); 2) monitoring the progression of the residents undergoing a number of different programs in each clinical department; and 3) assisting in the creation of a remediation program for residents in difficulty. As in other countries, the residency program directors are key persons to identify and support residents in difficulty because they are attentive to the prevalence and characteristics of the residents in difficulty in their own departments.

### Participants

Participants in the study were all residency program directors (*n* = 157) across all specialties appointed in the northern postgraduate medical training region in Denmark.

### Ethics statement

Permission for data extraction and handling was sought and approved by the Danish Data Protection Agency (J.nr. 2013-41-1794), and due to the non-biological and non-sensitive nature of the data it is exempted from the rules of the Danish Research Ethics Committee.

### E-survey and database study – comparing two groups of residents

The e-survey study consisted of a questionnaire that was distributed to all 157 residency program directors in the above-mentioned region. The questionnaire was divided into three parts. In the first part, the residency program directors were asked about department demographics and how many residents in difficulty were identified in their departments in 2012. In the second part, the residency program directors were asked to respond to a sequence of questions regarding their most recent experience with a resident in difficulty. The questions concerned: 1) demographic characteristics of the resident in difficulty (age, gender, nationality, educational background, postgraduate educational level, and specialty); 2) the residency program directors’ perception of the resident’s behaviour; and 3) the residency program directors’ perception of contributory causes to the resident’s difficulties. Similar to the studies of Tabby and colleagues [6] and Yao and Wright [1], we distinguished between the behaviours and the causes related to the resident’s difficulties. In the third part of the questionnaire, the residency program directors were asked to identify their clinical departments’ courses of action for the most recent residents in difficulty. The questions in part two and three were designed as a list of items from which each residency program director could choose one or more items. The lists of items were generated from existing studies on program directors’ perceptions of problem residents and residents in difficulty [1, 6] and referred to known behaviours and causes connected with residents in difficulties [13, 20, 32, 33].

In order to be able to compare residents in difficulty with the background population (all residents in the same region as the residents in difficulty), we collected database data on demographics of all residents in the region for the calendar year 2012. We extracted information from the database ‘Evaluer.dk’ on selected variables such as age, gender, and specialty. Hence, in the following paragraphs of the paper we refer to two groups: 1) the population in focus: the residents in difficulty in 2012 (the e-survey data); and 2) the background population: all residents in 2012 (the database data). The differences in distributions in the two groups (residents in difficulty in 2012 versus all residents in 2012) were tested using either a Chi-squared test (*Χ*^2^) or Fisher’s exact test statistics.

### Focus group interviews

A qualitative study consisting of focus group interviews [34] with residency program directors was completed in order to produce an in-depth understanding of the topics in the questionnaire. We included participants on the basis of three criteria in order to obtain a representative cross section of program directors: gender, geographical diversification (regional hospitals and university hospitals respectively), and specialty diversification. Three focus group interviews were conducted. Each group included six to eight residency program directors. All participants gave their informed written consent prior to the interviews. In our application of the focus group as a method for data collection, we emphasized the relatively ‘non-hierarchical’ relationship in the conversations between interviewer and participants. The conversations were conducted as semi-structured interviews with the help of an interview guide, which consisted of four overall themes based on the questionnaire in the e-survey study: 1) behaviours and characteristics of residents in difficulty compared to successful residents; 2) timing of the difficulties (early or late in the course of postgraduate medical education); 3) perceived contributory causes to the resident’s difficulties; and 4) types of intervention. The interviews (80–100 min per interview) were conducted in Danish, moderated by two of the authors, audio-recorded, and transcribed verbatim. To preserve anonymity, participants and locations are provided with pseudonyms in the presentation of the results.

The analysis of the data employed qualitative content analysis [35]. The first author read and categorized the transcribed focus group interviews. The four themes in the interview guide served as orientation for inductive coding using QSR NVivo (version 10). The first author identified six codes and 27 subcodes in an iterative process of integrating each new code or subcode in the analyses of already coded text. The codes and subcodes were further discussed and approved by the fourth and fifth author. This process ensured that themes were comparable across the three focus groups. The six codes were: code 1) behaviours and characteristics of successful residents in postgraduate medical education; code 2) behaviours and characteristics of residents in difficulty; code 3) examples of actual cases illustrative of residents in difficulty; code 4) contributory causes to the residents’ difficulty; code 5) types of intervention; code 6) emotional strains on the residency program director.

## Results

Firstly, we will present the results from the e-survey and the database studies. Secondly, we will present the results from the analysis of the focus group interviews. Due to the aim of this article, which is to examine the prevalence and characteristics of residents in difficulty in Denmark, we will limit the presentation of results from the focus group interviews to the first four codes. A more robust analysis and discussion of the two latter codes: 5) the different types of interventions applied in the remediation of a trainee in difficulty and 6) the emotional strains (such as self-doubt and lack of collegial support) on the residency program director, is located elsewhere. Finally, we will compare the quantitative and qualitative data.

### Prevalence and characteristics

Of the 157 invited respondents, 115 (73.2 %) completed the e-survey. The respondents reported the prevalence of residents in difficulty in the region to be 6.8 % (138/2,041) in 2012. There were no statistically significant differences in the prevalence of residents in difficulty by type of specialty, when specialty was categorized into four main groups – internal medicine, surgery, general practice, other (e.g. pharmacology, genetics, microbiology, pathology etc.). The respondents saw between zero and nine residents in difficulty in their departments in 2012, with a median of one problem resident per department. Whilst there were no differences in the gender distribution between residents in difficulty and the background population (Table 1), residents in difficulty were somewhat older and more likely to be at advanced levels of training compared to the background population (Table 1). In the subgroup of specialist residents, having an international medical degree was three times more common in the group with difficulties than in the background population (Table 1). The chance of being categorized as a resident in difficulty was higher for residents in university hospital departments than for residents in regional hospitals (OR = 2.50 [1.66–3.76]; Table 2).

**Table 1:** Residents in difficulty versus all residents in the region in 2012

Variable	Residents in difficulty (*n* = 133)	All residents (*N* = 2399)	Test	*p*-value
Gender				
Female	76 (57 %)	1445 (60 %)	*Χ* ^2^ = 0,5018	0.479
Male	57 (43 %)	954 (40 %)		
Total	133 (100 %)	2399 (100 %)		
Age (years)				
≤30	24 (18 %)	569 (24 %)	FET	0.005
31–35	58 (44 %)	948 (39 %)		
36–40	28 (21 %)	638 (27 %)		
≥41	22 (17 %)	244 (10 %)		
Missing	1 (0 %)	0 (0 %)		
Total	133 (100 %)	2399 (100 %)		
Level of training				
Basic	11 (8 %)	382 (16 %)	FET	0.004
Introduction	34 (26 %)	634 (26 %)		
Specialty	87 (65 %)	1383 (58 %)		
Missing	1 (1 %)	0 (0 %)		
Total	133 (100 %)	2399 (100 %)		
University				
National	50 (58 %)	1165 (84 %)	FET	0.000
International	34 (39 %)	180 (13 %)		
Missing/unknown	3 (3 %)	38 (3 %)		
Total (specialty residents)	87 (100 %)	1382 (100 %)		

*FET* Fisher’s exact test. Level of training: The first year in postgraduate training is ‘basic’ training (1 year), followed by ‘introduction’ to the specialty (1 year), and then finally by ‘specialty’ training (4–5 years depending on specialty). The questionnaire allowed respondents to describe up to five residents in difficulty. However, two respondents experienced six and nine residents in difficulty respectively, therefore only data from 133 of the total 138 residents in difficulty is available and presented above

**Table 2:** Residents in difficulty by type of department in 2012

Outcome	University hospital departments	Other departments	Total
Residents in difficulty	107 (8.8 %)	31 (3.7 %)	138
Other residents	1104 (91.2 %)	799 (96.3 %)	1903
Total	1211 (100.0 %)	830 (100.0 %)	2041

Pearsons *χ*2 = 20.3242, *p* = 0.000. University hospitals were defined as: Aarhus and Aalborg university hospitals, including the psychiatric departments in Risskov and in Aalborg university hospital. All other departments were considered non-university hospital departments

A total of 64 % (64/100) of the most recent residents in difficulty respondents had had experience with were identified as having difficulties within the first 3 months in the department, and 16 % (16/100) were identified as struggling even before their first day in the department. However, the difficulties were not acted upon by the departments until the third or fourth year in specialty training.

According to the respondents, the most common behavioural characteristics of residents in difficulty related to lack of competence in the leader/administrator role and in the professional role. More specifically, these residents had difficulty in completing tasks within a reasonable time frame; they did not take sufficient leadership in collaborative situations: they did not show an adequate understanding of their own role and abilities; and they did not act adequately in stressful situations (Table 3).

**Table 3:** Behavioral characteristics of the latest resident in difficulty (n_respondents_ = 100)

Role	Behavior characteristics	Number of respondents agreeing	Sum
Leader/administrator	Had difficulty with completing tasks within a reasonable time frame	48	96
	Did not take leadership in collaborative situations	48	
Other	Did not act adequately in stressful situations	53	81
	Disappeared while on duty/absent from work	28	
Collaborator	Did not function well in relations with colleagues and/or other personnel	36	77
	Was not constructive in collaborative situations	41	
Medical expert	Made many mistakes in clinical practice	26	69
	Could not transfer knowledge to clinical practice	43	
Communicator	Did not communicate with patients and/or relatives adequately and respectfully	25	63
	Was unable to explain/convey medical problems	38	
Professional	Did not act in accordance with ethical guidelines	7	60
	Did not show an adequate understanding for own role and abilities	53	
Scholar	Did not show an interest in acquiring new knowledge or skills	28	56
	Was not an active participant in the educational opportunities in the department	28	
Health advocate	Did not show an understanding of patients’ social and/or cultural backgrounds while giving advice	19	38
	Had difficulties with counselling and guidance	19	

Each respondent could assign multiple characteristics to the latest resident in difficulty

When asked about the perceived causes for the resident being in difficulty, the respondents generally tended to list personal attributes of the residents, such as insecure/nervous behaviour and lack of necessary motivation, as the main causes. Less frequent causes identified were lack of basic knowledge and skills, and stressors in the personal life of the resident (Table 4).

**Table 4:** Assigned causes for the latest resident being in difficulty (n_respondents_ = 100)

Category	Causes	Number of respondents agreeing	Average
Attributes	The resident was too insecure or nervous	41	41
	The resident was unable to receive constructive feedback	40	
	The resident did not poses the necessary will or competences to comply with the department’s medical standards	41	
Stressors	Stressors in the residents personal life situation	25	14
	Psychological or psychiatric health problems	15	
	Physical health problems	10	
	Substance abuse	3	
	Work-related stress and depression	17	
Education	The resident lacked in basic medical knowledge and/or clinical skills	36	16
	The resident did not receive adequate in-training feedback and/or supervision	12	
	The resident did not have sufficient opportunity to practice relevant procedures	4	
	The resident did not experience adequate social acceptance in the department	13	

Respondents were allowed to assign multiple causes

The types of interventions used to help residents in difficulty in the respondents’ departments are listed in Table 5. Intensification of dialog and formal meetings with the residents in difficulty were the most common types of intervention, followed by intensification of concrete clinical instruction, supervision, and feedback (Table 5). In the category ‘other’, the most common interventions used were: language programs, deceleration, and psychological interventions (Table 5).

**Table 5:** Remediation: What did you do in the department to help the latest resident in difficulty?(n_respondents_ = 100)

Remediation	n (respondents)
Extra talks with resident to clear up misunderstandings	66
Intensify supervisor meetings with resident	62
Intensify supervision/feedback in clinical situations	48
Concrete instruction in areas of incompetence	48
Other	31
Career guidance in relation to change/choice of specialty	30
Change the type of tasks	29
Temporary partial relief of duties	21
Assign a new supervisor	12

The respondents were allowed to assign more than one strategy as answer to the question

In addition to the general findings from the e-survey and database study, the focus group interviews unveiled another important layer of details about residents in difficulty.

### Personal and contextual characteristics

In total, 22 residency program directors (13 females and nine males; seven from regional hospitals and 15 from university hospitals; five from surgery, nine from internal medicine, and eight from other specialties) participated in the focus group interviews. We invited the residency program directors to compare behaviours and characteristics of residents in difficulty and successful residents. Similar to the results of the e-survey and database study, the residency program directors highlighted differences in personal attributes of residents in difficulty. Furthermore, the program directors described the successful residents in shorter sentences and with keywords, whereas the descriptions of residents in difficulty were longer and included more complex explanations concerning contextual characteristics of each resident’s workplace. The extracts below summarize the residency program directors’ descriptions of the successful residents (code 1: behaviours and characteristics of successful residents in postgraduate medical education) as persons who are willing to learn and are able to adapt to new circumstances and surroundings.

“They enjoy their work”; “They take responsibility for their own learning”; “They quickly become a part of the team” and “They easily readjust and adapt to new demands” (focus group 1).

“They are curious, investigative, and willing to learn” and “They have to be bold, because they need to cross their own comfort zone in order to learn something” (focus group 2).

“They are professional”; “They are able to communicate with everybody about everything, and they show that they are able to pick up new things” and “They have the will and the motivation to learn” (focus group 3).

In comparison, the residency program directors’ descriptions of the behavioural characteristics of residents in difficulty (code 2: behaviours and characteristics of residents in difficulty) were more complex; thus the analysis of code 2 resulted in seven subcodes that sum up the residency program directors’ accounts. Below, each of the seven subcodes are illustrated by an extract from the focus group interviews.

**Laying low or vanishing while on duty**: “Some residents hide while on duty; they lay low and stay anonymous for some time until they find out what’s going on here” (focus group 3).

**Poor patient communication skills**: “I gave feedback to some of the residents at the emergency course because their professionalism and their patient communication skills were poor: ‘Now, let’s bang the venous catheter into your arm’, one of them said, and also even more crude things. This is not the way to communicate with patients! But surprisingly, the residents were stunned by my feedback” (focus group 2).

**Inflated confidence**: “Soon we became aware that this resident had a quite different self-image and much grander thoughts about own skills and capacities than we had. And it took a long time before we managed to evaluate this problem. We discovered that the resident had been allowed to skim over his previous appointments without any consequences. Although the previous places of appointment have had some conversations with him, he wouldn’t comply with the suggested changes (focus group 2).

**Emotionally affected by the job**: “For some residents, the distinction between being a professional and being personal had disappeared. When you ask them, for instance, how they perceive the word empathy, some of the residents have not considered that the capacity to understand a person does not include being compassionate and emotional about this person. When you engage emotionally with your patients you risk acting inappropriately” (focus group 3).

**Cannot prioritize the daily tasks**: “Some residents try to keep several balls in the air, but they cannot complete anything, because they always take on new tasks. And then you discover that they have not managed to complete half of the tasks of the daily program, because they took on too many tasks” (focus group 3).

**Lack key competences and skills**: “You may have a problem in the department when a resident apparently complete the outpatient clinic with a large number of patients on schedule, but nobody really knows what is going in there…I think many colleagues have troubles about the dilemma figuring out exactly how skilled the resident is or if the resident is too hasty and slipshod in the outpatient clinic” (focus group 3).

**Lack of cooperative skills that are relevant in the local setting**: “[It has been difficult to cooperate with] a few residents with a background in other cultural settings where you don’t … [pause, silence] … where you have a power relation between doctors and nurses and other staff, so that for instance a doctor does not act on nurses’ instructions or on female doctors’ instructions for that matter (focus group 2).

In the focus group interviews, the residency program directors were offered the opportunity to explain their accounts by exemplifying specific cases of residents in difficulty. These cases (code 3: examples of actual cases illustrative of residents in difficulty) illustrated that most residents in difficulty exhibit several of the above-mentioned behavioral characteristics in different combinations. The described diversity and complexity of the residents in difficulty accentuates the fact that identifying one exact difficulty and designing adequate remediation is indeed perceived as a multifaceted challenge by the residency program directors we interviewed.

The presumed contributory causes of the difficulties (code 4: contributory causes to the residents’ difficulty) were many and diverse. Thus, the analysis of code 4 resulted in 10 subcodes that sum up the residency program directors’ accounts. The subcodes are related to personal attributes similar to the above-mentioned behavioural characteristics and to social, cultural, and/or organizational matters. For example, they include workplace-related traumatic episodes, illnesses (own or in the family), cultural differences (residents from other countries), a generation gap, and many short-term appointments in different departments during residency training. However, two of the ten contributory causes were mentioned numerous times in all focus group interviews and stood out as prime underlying reasons for residents ending up in difficulty. The extracts below illustrate these two causes:

**Cause 1:**

**Belated and inconsistent feedback on the resident’s inadequate performance**

“But it’s also much easier to just send the doctor back in the system to the next appointment. So, it is very hard for us to start this thing about a resident in difficulty, and in general, actually to terminate the resident’s training. So, everything calls for closing your eyes and letting time pass” (focus group 1).

“We are simply too nice and too neat, and it is inappropriate in this context because we do not dare tell the truth” (focus group 1).

“The question is: Does it become a threat to the resident if you say it early in the appointment?” (focus group 2).

“Well, it took 6 months before the residency program director dared mention that there could be a problem, and it’s really, really hard. We have *so* much trouble accepting that this is a resident that just cannot make it here” (focus group 3).

“And residents who are helpful and always get up and take the call, and always assume an extra call and make the outpatient clinic work smoothly – these residents are very useful for our system, but sometimes we (the senior doctors) don’t know the quality of their work or what is really happening in the outpatient clinic when we are not there. And then it can be difficult to argue for your concerns, if you are the only one saying that ‘I have a feeling that there is something wrong professionally with that resident’. Then the collective acknowledgements are delayed, because the resident in difficulty is nice to have in the ward [because the person is helpful] regardless of his or her lack of competencies” (focus group 3).

**Cause 2:**

**The perceived culturally rooted priority of efficient patient care before education in the workplace**

“Being a culture bearer (as we are) may also be in this respect to ensure to create enough time for training. Who would otherwise create it than ourselves? The problem is that we may not just agree on how much time for training, because we might consider our own and our department’s interests to be more important. So when you go to meetings, then you’ll hear sometimes … and it is not because it is only surgeons, but it can often be surgeons, and they do not come to our supervisory meetings because ‘I don’t have [swear word] time, I am supposed to [swear word] operate, and I am *not* supposed to [swear word] educate people, I’ll join you when I’m finish operating’” (focus group 1).

“But changing a culture, that’s really what the problem is. The departments have a huge responsibility for those so-called ‘residents in difficulty’. The residents all have high GPA’s from high school, they have undergone the longest of the hardest education at university, so it’s not real jerks we receive from medical school, but they may have some personality weaknesses and infirmities, which means that they must be guided along the way” (focus group 2).

“If the surgical training, for instance, is not sufficiently systematic, then the residents risk ending up in difficulty without necessarily self-inducing the situation. You have to know how the education system in that surgical department is running, otherwise remediation does not help, because if things [training and educational activities] are too sloppy in a department, you will come up with a plan that runs just as sloppily, and nobody is helped by that” (focus group 3).

Both of these causes (cause 1 and cause 2) relate to sociocultural challenges that involve workplace culture including social relationships among colleagues, ingrained habits in the department, and cases of consultants de-emphasizing their educational responsibilities.

## Discussion

Problem residents are found across most specialties in different countries at a prevalence of about 3–10 % [2, 4–6, 24]. In our study we found that 6.8 % of doctors in postgraduate medical education struggle to comply with educational requirements and occupational adaptation. Thus the prevalence of residents in difficulty in the northern postgraduate medical training region in Denmark is similar to the prevalence in other countries regardless of specialty. Clearly, even a modest prevalence of residents in difficulty is a concern, because every single struggling resident risks posing a liability to patients’ healthcare and bearing considerable personal costs [7]. In addition, our study indicated that sociocultural problems, in terms of belated feedback on inadequate performance and a perceived priority of efficient patient care before education in the workplace, may explain why some residents end up in difficulty. Similar findings were reported in comparable studies [1, 4, 17]. For example, Dupras and colleagues [4] addressed an interesting issue concerning local conditions in terms of feedback culture in resident training and its potential effect on each resident’s difficulty and subsequent remediation. This theme is also prevalent in our study, and we will argue that in the daily routines and busy reality of doctors, the observable markers (such as personal attributes and behaviors) are not always distinguished from the causes, and thus the local feedback culture – as well as research on residents in difficulty – tend to fault the resident in difficulty and not the local conditions of residency training. However, when the residency program directors in our study were offered the opportunity and time to discuss and reflect upon this paradox – as established in the focus group interviews – they voiced more nuanced social and cultural problems as contributing causes to resident difficulty.

### Individual level and system level – two sides of the same coin?

The findings seemed to disclose a discrepancy between, on the one hand, the reported behavioural problems, being a matter of the expected social relationship between the doctor and his/her workplace environment, and, on the other hand, the strong emphasis on individual attributes as the explaining causes of the difficulties. Previous studies also emphasize individual attributes as the explaining causes of difficulties. For example, Long [25] listed seven early signs of residents in difficulty: among them the disappearing act, low work rate, bypass syndrome, and insight failure. Also, a longitudinal retrospective review of resident records from the University of Toronto Faculty of Medicine’s Board of Examiners for Postgraduate Programs [5] showed that the residents had difficulties with an average of 2.6 of the CanMEDS Roles, with highest frequencies of Medical Expert (85 %), Professional (51 %), Communicator (49 %), Manager (43 %), and Collaborator (20 %). Unlike most studies on residents in difficulty, but similar to our study, this study compiled data on residents in difficulty from all residency programs across specialties. Similar to our findings, the authors did not report any differences between the specialties. The authors concluded that most residents in the study had multiple areas of weakness and that a standardized reporting template based on the CanMEDS framework is a useful tool for identifying and categorizing the areas of resident weakness. Also in a longitudinal retrospective review of all letters, e-mails, and incident reports for general surgery residents from 1995 to 2005, Resnick and colleagues [17] found that the most common complaints about resident behavior concerned unprofessional conduct (83 %). In our study, we also found that the lack of competence in both the role as leader/administrator and the professional role was perceived by the program directors as the most prevalent characteristic of residents in difficulty. The interview data, in particular, showed that the perception of these individual lacks of competence were context dependent and often caused by belated and inconsistent feedback on the concerned resident’s inadequate performance. The same trend was reported by Dudek and colleagues [36], who reported that clinical supervisors often do not fail students and residents even though they have judged their performance to be unsatisfactory. In their findings, the authors identified four major areas of the evaluation process that act as barriers to reporting a resident who has performed poorly: 1) lack of documentation; 2) lack of knowledge of what to specifically document; 3) anticipating an appeal process; and 4) lack of remediation options.

In addition, supervisors may feel that they lack sufficient skills to approach the resident in difficulty effectively, i.e. that they could be opening a can of worms which could potentially make things worse [7, 36, 37], or that the resultant emotional distress and self-doubt could be inimical to learning [4, 38].

Our study cannot determine the reason for the belated and inconsistent feedback on residents’ inadequate performance and unprofessional behavior. However, belated and inconsistent feedback may be caused by inadequate evaluation systems; for example, Adams and colleagues [24] showed that problems and concerns about unprofessional behavior among residents initially come to the program directors’ attention through personal communication, such as an e-mail or phone call from a faculty member, nurses or other residents, rather than through their program’s formal resident evaluation system. Hence, the program directors were frequently placed in the uncomfortable position of providing a recommendation for a resident about whom they have doubts, because the formal evaluation system regarding professional behaviour seems inefficient and inadequate to identify problem residents with unprofessional behaviour.

We must emphasize that identifying individual characteristics in a resident does not solve the problem of residents in difficulty in general. Hence, we agree with Tabby and colleagues [6]: “We do not recommend the use of this study as a tool to select future residents for neurology programs. Problem residents happen”. Or, in other words, identifying particular disturbing attributes in the individual resident and eventually excluding residents exhibiting these disturbing attributes does not per se prevent a system and a workplace culture under pressure due to high demands on patient care productivity and efficiency [29] from causing residents in difficulty. On the other hand, early identification of residents in difficulty is of course important and necessary for initiation of remediation and support of these residents [7].

### The workplace culture – a sociocultural challenge

Considering all the findings in our study, residents in difficulty seem to be a sociocultural challenge that exists because of immanent processes and complications at an individual level (in the personal sphere) and a systemic level (in the workplace culture) respectively. As shown in our study, two contributory causes were mentioned numerous times in all focus group interviews and stood out as prime underlying reasons for residents ending up in difficulty: belated and inconsistent feedback on the residents’ inadequate performance and the perceived culturally rooted priority of patient care before education in the workplace. These two causes seem to be embedded in a workplace culture and at a system level. Thus they demand a sociocultural rather than an individualistic approach in order to be explained and eventually solved. However, research on the perceived underlying causes of residents in difficulty mainly concern personal attributes of the resident, whereas the workplace culture is rarely examined. Only a few previous studies report that sociocultural challenges at a system level are prior causes of individually based causes of difficulty in medical education [29, 32]. In a study on program directors’ willingness to communicate their concerns about unprofessional behaviour among residents, Adams and colleagues [24] point out an important process in the workplace culture in the health care system – the manners of teaching and transmitting professional attitudes:

When we teach, in addition to the knowledge and skills we intend to convey, we also transmit a vast array of behaviours, beliefs, and attitudes we never intended to share, or even recognized we were imparting—the so-called “hidden curriculum”. […] Nevertheless, it is important to recognize that trainees and physicians in all training and practice settings at times display unprofessional behaviors, including those designated as role models.

Most interestingly, the authors found that the majority of program directors felt that their efforts at remediation aimed at the individual level of the resident were only somewhat successful. An important question left unanswered by the abovementioned studies is the option of remedying ‘a workplace culture in difficulty’, since these studies did not examine residents’ workplace cultures. Our results indicate that program directors were well aware of the influence of disturbing workplace cultures on the socialization of residents. Thus our results support a most interesting ethnographical study by Szymczak and Bosk [29] that demonstrate ways in which workplace cultures in the healthcare system ‘teach’ residents the social norm of efficiency and high workloads and at the same time ‘teach’ residents to tolerate seemingly intractable systemic problems regardless of the resident’s individual learning needs. In fact, the residents in Szymczak and Bosk’s study depicted themselves primarily in opposition to “the system”. The program directors in our study reported a similar experience. From this background we suggest that a “system” like this risks posing a liability to residents’ education. From a sociological perspective, the French sociologist Pierre Bourdieu [39] advocated that the *doxa* (from Greek: common belief) of a system, that is the common sense “accepted by all as self-evident” and the dominant social norm (often preverbal and taken for granted) in a system such as the healthcare system, appears to resist change even if change seems to be required, because *doxa* is closely connected to powerful positions and intractable traditions in the system. If this is the case, and if we intend to prevent systems from contributing to causes that generate residents in difficulty, then the remediation of ‘a workplace culture in difficulty’ seems as imperative as the remediation of individual residents in difficulty.

Our study has limitations. The main weaknesses include the limited population in the study. We only included program directors from one of three postgraduate medical training regions in Denmark, and although we had a relatively high response rate (73.2 %), we cannot conclude that our results represent Danish program directors’ perceptions of residents in difficulty in general. Also, the limited number of focus group interviews may pose a restriction to our aim to grasp the general perceptions among program directors across different specialties and regions in Denmark. However, we consider data gathered in this way to be worthwhile to pursue and include in examinations of respondents’ beliefs and experiences of complex phenomena, such as residents in difficulty.

Despite these limitations, out study provides important insights into the dual nature of difficulties for residents in the healthcare system: while personal attributes and behavioural characteristics of the individual resident may serve as observable markers in the process of identifying a resident needing remediation and support, sociocultural problems in terms of belated feedback on inadequate performance and a perceived priority of efficient patient care before education in the workplace may well explain why some residents end up in difficulty.

## Conclusions

The results of this mixed method study showed the prevalence of residents in difficulty was 6.8 %. We found no statistically significant differences in the prevalence of residents in difficulty by type of specialty. According to the residency program directors, the most common behavioural characteristics of residents in difficulty are related to lack of competence in both the role as leader/administrator and the professional role. The results also showed that two important themes related to the workplace culture of the resident in difficulty were belated and inconsistent feedback on the resident’s inadequate performance, and the perceived culturally rooted priority of efficient patient care before education in the workplace. These two themes were emphasized by the program directors as the primary underlying causes of the residents’ difficulty. More work is needed in order to clarify the link between, on the one hand, observable markers of residents in difficulty and, on the other hand, immanent processes and logics of practice in a healthcare system. From our perspective, further sociological and pedagogical investigations in educational cultures across settings and specialties could inform our understanding of and knowledge about pitfalls in residents’ and doctors’ socialization into the healthcare system.

## Abbreviations

QSR NVivo: is a computer software package for qualitative data analysis. It is produced by QSR International
GPA: is grade point average and is the student’s average score of all grades from all current classes
CanMEDS: is a framework for medical education that sets standards for essential competencies expected of physician specialists in Canada. The framework is applied in the Danish healthcare system

## Footnotes

1 In this paper we use the term *resident in difficulty* as a generic term for problem resident, trainee in difficulty, problem learners, struggling trainees and struggling residents.
